# Weekday Sleep Duration and Perceived Restorative Sleep, but Not Dietary Intake, Are Associated with Lower Skin Autofluorescence in Japanese Early Adolescent Girls: A Cross-Sectional Study

**DOI:** 10.3390/nu18091377

**Published:** 2026-04-27

**Authors:** Toshiyuki Kohri, Nozomi Okamoto, Chiho Myojin, Masako Kawanishi, Yumika Makita, Mako Yamamoto, Yuko Higashine, Mariko Nakamoto

**Affiliations:** 1 Department of Clinical Nutrition and Dietetics, Faculty of Clinical Nutrition and Dietetics, Konan Women’s University, 6-2-23 Morikita-machi Higashinada-ku, Kobe 658-0001, Japan; yamamoto@konan-wu.ac.jp (M.Y.); higashine@konan-wu.ac.jp (Y.H.); 2 Department of School Psychology, Developmental Science and Health Education, Hyogo University of Teacher Education, 942-1 Shimokume, Kato 673-1494, Japan; onozomi@hyogo-u.ac.jp; 3 Department of Food and Nutrition, Faculty of Agriculture, Kindai University, 3327-204 Nakamachi, Nara 631-8505, Japan; myojin@nara.kindai.ac.jp (C.M.); masako_kawanishi@mist.ocn.ne.jp (M.K.); yumika.makita@nara.kindai.ac.jp (Y.M.); 4 Department of Public Health and Applied Nutrition, Institute of Biomedical Sciences, Tokushima University Graduate School, 3-18-15 Kuramoto-cho, Tokushima 770-8503, Japan; nakamoto@tokushima-u.ac.jp

**Keywords:** advanced glycation end product, AGE, skin autofluorescence, early adolescence, dietary intake, sleep duration, perceived restorative sleep

## Abstract

Background: Advanced glycation end products (AGEs) accumulate in tissues with age and are associated with the risk of chronic diseases. However, evidence regarding lifestyle factors related to AGE accumulation in healthy adolescents is limited. The aim of this study was to explore dietary and lifestyle factors that may attenuate tissue AGE accumulation, using skin autofluorescence (SAF) as a noninvasive proxy marker, in healthy Japanese early adolescent girls. Methods: This cross-sectional study included 315 first-year junior high school girls aged 12–13 years from a private school in Japan. SAF was measured on the volar forearm using an AGE Reader MU. Dietary intake was assessed using a validated brief diet history questionnaire (BDHQ-15y). Lifestyle factors, including weekday sleep duration, were assessed using a self-administered questionnaire. Health-related variables (including weight-loss dieting) were also collected. Associations between SAF and each factor were analyzed using generalized linear models and nonparametric tests, with multivariable adjustment for potential confounders. Results: The mean SAF was 1.06 ± 0.13 arbitrary units. No significant associations were observed between SAF and health-related characteristics, nutrient intakes, or major food-group intakes. Longer weekday sleep duration was significantly associated with lower SAF (*p* for trend = 0.019) and remained significant after multivariable adjustment (*p* for trend = 0.018). A similar association was observed for better perceived restorative sleep (*p* for trend = 0.033; adjusted *p* for trend = 0.048). Conclusions: In healthy early adolescent girls, longer weekday sleep duration and better perceived restorative sleep were associated with lower SAF, whereas dietary intake was not. Given the largely irreversible age-related accumulation of AGEs, promoting healthy sleep during adolescence may help attenuate AGE accumulation early in life and reduce long-term AGE-related disease risk. Prospective studies with more detailed dietary assessments are needed to clarify dietary influences and confirm temporality.

## 1. Introduction

Recently, advanced glycation end products (AGEs) have attracted increasing attention as risk factors for lifestyle-related diseases. Prospective studies and meta-analyses have shown that higher levels of AGE accumulation markers, such as skin autofluorescence (SAF) and circulating AGE concentrations, are associated with an increased risk of type 2 diabetes, cardiovascular disease, and mortality in both the general population and patients with diabetes [1,2].

AGEs are a heterogeneous group of compounds formed through non-enzymatic reactions between carbonyl groups of reducing sugars (e.g., glucose) and amino groups in proteins, lipids, and nucleic acids [3]. This process proceeds via early glycation products (Schiff bases and Amadori products) and subsequent oxidation and dehydration reactions, ultimately leading to stable AGE formation [3].

Two major pathogenic mechanisms of AGEs have been proposed. First, AGE-mediated cross-linking alters the three-dimensional structure and physicochemical properties of proteins and lipids, thereby promoting tissue damage and aging-related changes. Second, AGEs bind to the receptor for AGEs (RAGE) on the cell surface, activating oxidative stress and inflammatory signaling pathways such as NF-κB and thereby amplifying chronic inflammation [4]. Another important characteristic of AGEs is their resistance to metabolic degradation and clearance, resulting in progressive tissue accumulation with age [5]. This age-related accumulation is more pronounced in middle-aged and older adults and has been implicated in the onset and progression of cardiovascular disease, diabetes, and its complications, as well as neurodegenerative disorders and malignancies [1,6].

AGE accumulation in the body may result from two major sources. One proposed source is intestinal absorption of exogenous AGEs from AGE-rich foods; foods subjected to high-temperature processing and cooking are thought to be important dietary sources of AGEs [7]. Epidemiological evidence is limited; however, ultra-processed foods have been proposed as important dietary sources of AGEs because industrial processing often involves high heat, and studies have begun to examine associations between ultra-processed food intake and tissue AGE markers [8]. The second source is endogenous AGE formation. In vivo, proteins are continuously exposed to glycation, and chronic hyperglycemia and increased oxidative stress promote endogenous AGE formation and tissue accumulation [4]. Dietary and lifestyle habits influence blood glucose levels and oxidative stress and are therefore expected to contribute to inter-individual differences in endogenous AGE generation. However, epidemiological studies examining associations between lifestyle factors, such as dietary habits and sleep status, and AGE accumulation in healthy populations remain scarce. In particular, insufficient or poor-quality sleep has been reported to induce sympathetic overactivity, increased insulin resistance, and elevated oxidative stress [9], which may promote AGE formation and tissue accumulation.

Given the age-related increase in tissue AGE accumulation [5], strategies to prevent AGE accumulation should be implemented not only in adulthood but also at earlier life stages. A non-invasive device (AGE Reader MU; DiagnOptics, Groningen, Netherlands) was developed to assess subcutaneous AGE accumulation by measuring SAF [10]. Because this device enables noninvasive assessment, it is suitable for studies in children and adolescents. To date, however, only two epidemiological studies have investigated associations between AGE accumulation and lifestyle- or clinical-related factors in healthy pediatric populations [11,12]: Köchli et al. reported that higher physical fitness was associated with lower SAF in children, whereas Nagahara et al. identified clinical factors, including family history of diabetes mellitus, associated with SAF in Japanese schoolchildren. To our knowledge, no such study has focused on healthy adolescents. Adolescence is a developmental period characterized by concurrent physical growth and psychosocial development, with particularly pronounced endocrine changes in girls. Because dietary and lifestyle habits formed during this period are likely to persist into adulthood, they may substantially affect long-term health outcomes.

This cross-sectional study targeted first-year female junior high school students in Japan. We aimed to examine associations of dietary and lifestyle habits with AGE accumulation levels by assessing SAF and to explore modifiable factors associated with lower SAF.

## 2. Materials and Methods

### 2.1. Participants and Ethical Approval 

Participants were first-year students enrolled in a private girls’ junior high school (School A) in Hyogo Prefecture, Japan, during the academic years 2023–2025. Eligible participants were healthy girls without diabetes or other chronic diseases. For the 2023 (*n* = 186), 2024 (*n* = 180), and 2025 (*n* = 168) cohorts, the study purpose and procedures were explained orally and in writing, and the students were invited to participate voluntarily. Parents and guardians were informed of the purpose and methods of the study. Written informed consent was obtained from 109 students in 2023, 98 in 2024, and 146 in 2025. Students who were absent on the survey day and those who declined to participate in the assessment were excluded. The final analytical sample comprised 97 students in 2023, 82 students in 2024, and 136 students in 2025 (total *n* = 315).

This study was approved by the Ethics Committee of Konan Women’s University (approval no. 202204, 30 May 2022), and was conducted from mid to late June of each academic year.

### 2.2. Anthropometric Measurements

Data were obtained from the most recent anthropometric records of first-year students in June for each school year. Body weight was measured to the nearest 0.1 kg and standing height to the nearest 0.1 cm while participants wore light clothing. Body mass index (BMI; kg/m^2^) was calculated as weight (kg) divided by height squared (m^2^).

### 2.3. Measurement of SAF

SAF was measured non-invasively using an AGE Reader MU (DiagnOptics Technologies, Groningen, Netherlands) and was reported in arbitrary units (AU). The detailed principles have been described previously [10]. Briefly, some AGE compounds are autofluorescent. The AGE Reader MU illuminates a 1 cm^2^ skin area with excitation light (300–420 nm) and detects autofluorescence emitted by multiple AGEs accumulated at a depth of approximately 1 mm in the dermis. Autofluorescence was defined as the average fluorescence per nm over the full emission spectrum (420–600 nm) and was expressed as a ratio of the average fluorescence per nm over the 300–420 nm range. The AGE Reader MU cannot detect non-fluorescent AGEs. However, because quantitative correlations between fluorescent and non-fluorescent AGEs have been reported [10], SAF values can serve as a proxy for total AGE accumulation.

Measurements were performed according to the device protocol. The volar side of the right forearm was used as the measurement site. The skin was cleaned with alcohol-free wet wipes to remove sunscreen or other surface contaminants and then fully dried using a small fan. The mean of three measurements was used as the SAF value in the analyses.

### 2.4. Dietary Assessment

Habitual intake of total energy, nutrients, and food groups was assessed using a brief-type self-administered diet history questionnaire modified for school students (BDHQ-15y). Its validity has been demonstrated by comparison with 8-day weighed dietary records [13]. The BDHQ-15y assessed dietary intake over the preceding month for 67 food items. Based on the reported consumption frequency of these food items and the BDHQ-15y calculation program, estimated daily intakes of energy, protein, fat, carbohydrate, and other nutrients were calculated. The questionnaire was distributed to students, and parents/guardians were asked to assist with its completion.

### 2.5. Self-Administered Lifestyle-Related Questionnaire

Participants completed a self-administered questionnaire on the survey day. Four-point scale items were coded as follows:

- Breakfast frequency (days/week): 1 = every day; 2 = 5–6 days; 3 = 3–4 days; 4 = ≤2 days.
- Fullness at meals: 1 = always eats until full; 2 = sometimes eats until full; 3 = eats until about 80% full; 4 = eats until less than 80% full.
- Main timing of snack intake: 1 = immediately after returning home; 2 = before dinner; 3 = after dinner; 4 = before bedtime.
- Bowel movement frequency (days/week): 1 = 1–2 days; 2 = 3–4 days; 3 = 5–6 days; 4 = every day.
- Subjective sleep quality (feeling rested upon waking during the past month): 1 = very good; 2 = good; 3 = not very good; 4 = not rested at all.
- Exercise frequency (days/week, excluding school classes): 1 = 6–7 days; 2 = 3–5 days; 3 = 1–2 days; 4 = <1 day.

Subjective sleep quality was based on the concept of “feeling sufficiently rested by sleep” in the Ministry of Health, Labour and Welfare (MHLW) Sleep Guidelines for Health Promotion 2023 [14]. Weekday sleep duration was calculated from self-reported mean bedtime and wake-up time and categorized into quartiles: 1 = ≤7.0 h; 2 = >7.0 to ≤7.75 h; 3 = >7.75 to ≤8.33 h; 4 = >8.33 h. Health-related variables (menarche, food allergy, hay fever, and weight-loss dieting) were assessed dichotomously (yes/no).

### 2.6. Statistical Analysis

All statistical analyses were performed using SPSS Statistics version 30. Participant characteristics are presented as means ± standard deviations. Normality was assessed using the Kolmogorov–Smirnov test. Because SAF was not normally distributed, nonparametric tests and generalized linear models (GLMs) were used.

For health-related variables, participants were compared between groups defined by a median split of BMI (≤median vs. >median) and by dichotomous status variables (menarche, food allergy, hay fever, and weight-loss dieting; yes vs. no). Between-group differences in SAF were evaluated using the Mann–Whitney U test, and results are presented as medians (interquartile ranges) of SAF.

Associations of SAF with nutrient intakes, food-group intakes, and weekday sleep duration were evaluated using GLMs with SAF as the dependent variable, assuming a gamma distribution with a log link function. Participants were categorized into quartiles according to nutrient intakes, food-group intakes, or sleep duration. To test linear trends across categories, quartile categories were treated as an ordinal term (1–4) in the GLMs. Estimated marginal means of SAF with 95% Wald confidence intervals are presented for each category. Linear trends for four-point lifestyle questionnaire items were analyzed similarly. Trend tests were also performed after adjustment for potential confounders; for brevity, adjusted results are presented only for factors with significant linear trends.

SAF_1_: SAF adjusted for energy intake and available carbohydrate intake.

SAF_2_: SAF adjusted for energy intake, available carbohydrate intake, BMI, menarche status, food allergy status, hay fever status, and weight-loss dieting status.

For the main timing of snack intake, 10 participants without snacking habits were excluded (*n* = 305 for this analysis). For egg intake, quartile classification was not feasible because many participants had identical values based on the BDHQ-15y algorithm; therefore, participants were divided into two groups by the median, and the Mann–Whitney U test was used. Statistical significance was set at *p* < 0.05 (two-sided).

## 3. Results

Participant characteristics, lifestyle factors, macronutrient, dietary fiber and salt-equivalent intakes are shown in Table 1. The mean age was 12.7 ± 0.3 years. The mean height and weight were 152.0 ± 5.5 cm and 42.1 ± 6.9 kg, respectively. The mean SAF value was 1.06 ± 0.13 AU. A previous report using the same device in 63 Japanese elementary school girls (10–12 years) showed an SAF value of 1.04 ± 0.14 AU [12], which was broadly comparable after accounting for the age differences.

Table 2 shows median SAF levels and interquartile ranges for participants categorized into two groups according to health-related characteristics. No significant differences in SAF were observed between BMI groups (≤median vs. >median; *p* = 0.226) or between groups categorized by menarche status (*p* = 0.134), food allergy status (*p* = 0.128), hay fever status (*p* = 0.573), or weight-loss dieting status (*p* = 0.102).

Table 3 presents the estimated marginal means of SAF and their 95% Wald confidence intervals across four groups categorized by quartiles of energy or major nutrient intake. No significant linear trends in SAF were observed across the four groups for any nutrient: energy (*p* for trend = 0.285), available carbohydrate (*p* for trend = 0.520), protein (*p* for trend = 0.421), fat (*p* for trend = 0.390), vitamin B_1_ (*p* for trend = 0.611), vitamin B_2_ (*p* for trend = 0.858), vitamin C (*p* for trend = 0.396), retinol activity equivalents (*p* for trend = 0.510), calcium (*p* for trend = 0.652), salt-equivalent (*p* for trend = 0.907), and dietary fiber (*p* for trend = 0.283). These trends remained non-significant after adjustment for energy intake and/or available carbohydrate intake and after further adjustment for BMI, menarche status, food allergy status, hay fever status, and weight-loss dieting status.

Table 4 shows the group sizes, cutoff values, and estimated marginal means of SAF with 95% Wald confidence intervals across four groups categorized by quartiles of major food-group intakes. No significant linear trends in SAF were observed across the groups for any food group: cereals (*p* for trend = 0.909), legumes (*p* for trend = 0.118), meats (*p* for trend = 0.688), fish and shellfish (*p* for trend = 0.729), dairy (*p* for trend = 0.382), green and yellow vegetables (*p* for trend = 0.911), other vegetables (*p* for trend = 0.107), fruits (*p* for trend = 0.965), confectioneries (*p* for trend = 0.824), and beverages, including tea, coffee, and soft drinks (*p* for trend = 0.639). These trends remained non-significant after adjustment for energy intake and available carbohydrate intake and after further adjustment for BMI, menarche status, food allergy status, hay fever status, and weight-loss dieting status.

For egg intake, participants were divided into two groups based on the median intake; however, no significant between-group difference in SAF was observed (*p* = 0.145).

Table 5 shows the results for participants categorized into four groups according to their responses to each lifestyle questionnaire item. A significant negative linear trend was observed between weekday sleep duration and SAF, such that longer sleep duration was associated with lower SAF (*p* for trend = 0.019). This trend remained significant after adjustment for energy intake and available carbohydrate intake (SAF_1_, *p* for trend = 0.017) and after additional adjustment for BMI, menarche status, food allergy status, hay fever status, and weight-loss dieting status (SAF_2_, *p* for trend = 0.018).

In addition, a significant negative linear trend was found between subjective sleep quality (feeling rested upon waking) and SAF, such that better sleep quality was associated with lower SAF (*p* for trend = 0.033). This trend remained significant after adjustment for energy intake and available carbohydrate intake (SAF_1_, *p* for trend = 0.041) and after additional multivariable adjustment for BMI, menarche status, food allergy status, hay fever status, and weight-loss dieting status (SAF_2_, *p* for trend = 0.048).

A significant positive linear trend was observed between exercise frequency and SAF, such that a higher exercise frequency was associated with higher SAF (*p* for trend = 0.021). This trend remained significant after adjustment for energy intake and available carbohydrate intake (SAF_1_, *p* for trend = 0.019) and after additional multivariable adjustment for BMI, menarche status, food allergy status, hay fever status, and weight-loss dieting status (SAF_2_, *p* for trend = 0.019).

No significant linear trends in SAF were observed across the four groups for the other lifestyle questionnaire items: breakfast frequency (*p* for trend = 0.121), fullness at meals (*p* for trend = 0.958), timing of snack intake (*p* for trend = 0.546), and bowel movement frequency (*p* for trend = 0.951). These trends remained non-significant after adjustment for energy intake and available carbohydrate intake and after further adjustment for BMI, menarche status, food allergy status, hay fever status, and weight-loss dieting status.

## 4. Discussion

In this study, we examined modifiable dietary and lifestyle factors associated with AGE accumulation in healthy early adolescent girls using SAF as a noninvasive proxy marker. To the best of our knowledge, this is the first study to examine both dietary intake and multiple lifestyle factors, including sleep-related variables, in relation to SAF in this population. The main findings indicate that longer weekday sleep duration and better perceived restorative sleep are associated with lower SAF, whereas nutrient and major food-group intakes were not. These findings suggest that healthy sleep habits may help attenuate AGE accumulation early in life.

During adolescence, a delayed circadian phase tends to postpone bedtime, and on school days, a mismatch with early school start times often leads to shortened sleep duration and chronic sleep insufficiency [15]. In a randomized crossover study, Klingenberg et al. reported that in 21 healthy adolescent boys, three consecutive nights of short sleep (4 h/night) versus long sleep (9 h/night) increased the homeostatic model assessment of insulin resistance (HOMA-IR) by 65%, indicating reduced insulin sensitivity even after short-term sleep restriction [16]. Spiegel et al. further showed that experimental sleep debt impairs glucose tolerance and shifts the sympathovagal balance toward sympathetic predominance [17]. These findings support biological pathways through which sleep loss may increase glucose levels. In an observational study, Matthews et al. reported that shorter sleep duration may be associated with higher HOMA-IR in 245 healthy high school students (mean sleep duration, 7.4 h) [18]. In addition, Rodrigues et al. found that sleeping <8 h/night (vs. ≥8 h/night) was associated with lower insulin sensitivity in 81 adolescents aged 10.0–19.9 years [19]. Collectively, a sleep duration of <8 h in adolescence may promote sympathetic predominance and reduce insulin sensitivity, predisposing adolescents to hyperglycemia and potentially facilitating AGE formation and accumulation. Consistent with this framework, in our study, the shortest sleep-duration quartile (≤7.0 h) tended to show higher SAF (Table 5), possibly reflecting a sustained internal milieu favoring AGE generation and deposition. Given that SAF is a proxy marker of tissue AGE accumulation, these findings suggest that sleep duration and sleep quality may influence glycation processes. This interpretation is supported by a recent cross-sectional study in healthy adults, which reported that shorter sleep duration was associated with higher SAF and that participants who slept < 6 h had significantly higher SAF than those who slept >8 h after multivariable adjustment [20].

We found a significant negative linear trend between subjective sleep quality (feeling rested upon waking) and SAF (*p* for trend = 0.033), with lower SAF in participants reporting better sleep quality (Table 5). Rawat et al. categorized 203 healthy young adults into evening, intermediate, and morning chronotypes and reported that the evening-type group had significantly worse sleep quality (Pittsburgh Sleep Quality Index [PSQI]), higher 2 h glucose levels during a 75 g oral glucose tolerance test, higher HOMA-IR, and shorter sleep duration than the other groups [21]. Galan-Lopez et al. also reported that among adolescents aged 13–16 years (900 boys and 817 girls), girls had significantly worse PSQI-assessed sleep quality than boys [22]. In our study, subjective sleep quality was defined according to the Japanese MHLW concept of “feeling rested upon waking,” and thus is not directly equivalent to the PSQI. Nevertheless, because the PSQI includes domains related to subjective sleep quality and sleep duration, these prior findings are broadly consistent with our results for girls. One plausible mechanism is that poor sleep quality, similar to insufficient sleep duration, promotes a hyperglycemic milieu, thereby accelerating non-enzymatic glycation and AGE formation.

In contrast, regarding physical activity, we found a positive linear trend: a higher exercise frequency was associated with higher SAF (Table 5). However, evidence supporting this association is limited. Wang et al. reported that in late adolescent females (aged approximately 18 years), SAF after 14 weeks of cheerleading practice (twice weekly; *n* = 21) was significantly lower than that in the controls (*n* = 25) [23], which is contrary to our findings. One possible explanation is measurement granularity, in which exercise frequency was assessed using a single self-reported item (days/week) without capturing intensity, duration, timing, or non-exercise physical activity. A methodological review of the AGE Reader noted that acute states associated with high-intensity activity (and potential glycoxidative stress) as well as higher subcutaneous melanin may elevate SAF readings [24]. Therefore, the positive association observed here should be interpreted cautiously, as it may reflect the unseparated effects of activity shortly before measurement or sun exposure from outdoor exercise, rather than chronic training adaptation.

No significant linear trends in SAF were observed for the other lifestyle questionnaire items, including breakfast frequency, fullness at meals, timing of snack intake, and bowel movement frequency (Table 5). However, these null findings should be interpreted cautiously. Several response categories were highly uneven or very small in size, which may have reduced statistical power. For example, most participants reported eating breakfast every day, whereas only a small number reported infrequent breakfast consumption, and only three participants reported snacking before bedtime. In addition, these questionnaire items were assessed using brief self-reported categories and may not have captured the qualitative or contextual aspects of these behaviors in sufficient detail. Therefore, the absence of significant associations in the present study does not necessarily indicate that these lifestyle factors are unrelated to AGE accumulation.

In this study, neither nutrient intakes nor major food-group intakes were significantly associated with SAF (Table 3 and Table 4). Previous studies, mainly in adults, have reported modest and inconsistent associations between habitual diet and SAF; for example, higher intake of meat or meat products has been associated with higher SAF, whereas higher cereal intake has been associated with lower SAF in adults [25]. Meanwhile, a recent study of university students found no significant association between ultra-processed food consumption and skin AGE levels [8]. These mixed findings may be partly attributable to methodological differences across studies. In the present study, the lack of associations for both nutrient and food-group intakes may also be partly explained by the young age of the participants. Because the participants were only 12–13 years old, the duration of dietary exposure may have been insufficient for differences in habitual diet to be reflected in tissue AGE accumulation as assessed by SAF. This limitation may be particularly important in adolescents, because SAF reflects cumulative tissue AGE accumulation, and dietary influences may therefore be harder to detect at earlier life stages, when lifetime exposure is shorter and inter-individual variability in SAF may be smaller. In addition, although dietary AGEs are strongly influenced by cooking and processing methods [26], our dietary assessment captured nutrient and food-group intakes over the preceding month rather than dietary AGE content itself. Consistent with this possibility, a large pediatric study (Italian I.Family Project) found no clear association between dietary AGE intake and urinary fluorescent AGEs in children and adolescents, although the biomarker used differed from SAF [27]. Therefore, our null findings for diet do not exclude potential dietary contributions to tissue AGE accumulation. Prospective studies in adolescents using repeated dietary assessment, including cooking methods and/or direct estimation of dietary AGE intake, together with concurrent measurement of tissue and circulating or urinary AGE markers, are warranted.

From a public health perspective, these findings may have practical implications for early prevention strategies. Because sleep duration and sleep quality are modifiable lifestyle behaviors, improving sleep habits during adolescence may represent a feasible and low-cost approach to mitigating early AGE accumulation. In particular, insufficient sleep among adolescents has been reported to be associated with school schedules, academic demands, and increasing evening electronic media use [28]. Therefore, promoting healthy sleep environments and sleep hygiene in both school and family settings may help maintain metabolic homeostasis and potentially reduce long-term risk of AGE-related chronic diseases.

### Limitations

First, the external validity of this study is limited because the participants were exclusively first-year girls from a single private junior high school in Japan. Therefore, the findings may not be generalizable to boys, adolescents from other school settings or regions, or populations with different socioeconomic or cultural backgrounds. Future studies including multiple schools and more diverse populations are needed to confirm the generalizability of the present findings. Nonetheless, according to the 2024 School Health Statistics Survey conducted by the Ministry of Education, Culture, Sports, Science and Technology (MEXT), the national mean height and weight of first-year junior high school girls were 152.3 cm and 44.4 kg, respectively, comparable to those of our cohort (Table 1). National data from the 2024 National Health and Nutrition Survey reported the following mean intakes in girls aged 7–14 years of 1799 kcal/day (energy), 67.3 g/day (protein), 60.5 g/day (fat), 238.6 g/day (carbohydrate), 16.3 g/day (dietary fiber), and 8.0 g/day (salt-equivalent). Although a direct comparison is limited because these estimates are based on 1-day dietary records, these values were not markedly different from our participants’ BDHQ-15y estimates (Table 1). In addition, the 2025 National Child Sleep Survey by the Hakuhodo Foundation Children’s Research Institute reported a mean weekday sleep duration of 7.9 h among junior high school students [29], broadly similar to our sample (Table 1). Taken together, our participants did not appear to deviate substantially from average Japanese first-year junior high school girls.

Second, we did not examine genetic or familial predispositions affecting SAF. Because information on family history of diabetes mellitus was not collected, we could not rule out the possibility that inherited or familial factors influenced SAF levels. Nagahara et al. reported that a family history of diabetes mellitus was associated with SAF in Japanese elementary school children [12], highlighting the need for further evaluation in future studies.

Third, because this was a cross-sectional study, temporal ordering could not be established, and causal inferences cannot be made. Reverse causation is therefore possible; poor or insufficient sleep may promote AGE formation and accumulation, while accumulated AGEs may, in turn, contribute to insomnia or poorer sleep quality. In addition, although we adjusted for several potential confounders, including energy intake, available carbohydrate intake, BMI, menarche status, food allergy status, hay fever status, and weight-loss dieting status, other relevant factors may not have been fully captured. For example, psychosocial stress, socioeconomic background, screen time, chronotype, and unmeasured aspects of physical activity or sun exposure may also have influenced SAF levels. Therefore, residual confounding cannot be excluded. Furthermore, the relationship between sleep and AGE burden may be bidirectional. This interpretation is supported by a recent study in Chinese adults showing that higher circulating AGE levels were associated with short sleep duration, poor sleep quality, excessive daytime sleepiness, and insomnia [30].

Fourth, although we observed no significant differences in SAF by menarche status (Table 2), sexual maturation factors (e.g., years since menarche and menstrual-related symptoms) may act as important confounders or effect modifiers of sleep quantity and quality [31], and may therefore influence SAF through changes in sleep. These factors should be examined in future studies.

Fifth, we were unable to assess cooking methods and therefore could not estimate dietary AGE intake [32]. In addition, we did not directly measure circulating AGE levels in blood.

Finally, we were unable to examine social jetlag. Because social jetlag in young people has been reported to be associated with depressive symptoms [33], it may also be related to AGE formation and accumulation, and should be addressed in future studies.

Despite these limitations, our findings suggest that, in healthy adolescent girls, ensuring sufficient sleep duration and improving sleep quality may help attenuate AGE formation and accumulation. This, in turn, may contribute not only to short-term improvements in glucose metabolism but also to a long-term reduction in the risk of AGE-related diseases. Given that AGE accumulation in tissues increases with age in a largely irreversible manner [5], these findings are meaningful. Prospective longitudinal studies are warranted to confirm temporal relationships and clarify the underlying mechanisms.

## 5. Conclusions

In healthy Japanese early adolescent girls, longer weekday sleep duration and better perceived restorative sleep (feeling rested upon waking) were independently associated with lower skin autofluorescence (SAF), a proxy marker of tissue AGE accumulation, after multivariable adjustment. In contrast, no significant associations were observed between SAF and nutrient intakes or major food-group intakes in this cohort; however, further studies with more detailed dietary assessment are warranted to clarify potential dietary influences. Exercise frequency showed a positive association with SAF, which should be interpreted cautiously because information on exercise intensity, duration, timing, and sun exposure was unavailable. Overall, these findings suggest that promoting healthy sleep during adolescence may help attenuate AGE accumulation early in life and potentially reduce long-term AGE-related disease risk. Prospective studies are needed to confirm temporality and underlying mechanisms.

## Figures and Tables

**Table 1:** Basic characteristics of 315 Japanese first-year junior high school girls.

	Mean	(SD)
Age (year)	12.7	(0.3)
Height (cm)	152.0	(5.5)
Weight (kg)	42.1	(6.9)
BMI (kg/m^2^)	18.1	(2.4)
SAF (AU)	1.06	(0.13)
Weekday sleep duration (h)	7.5	(0.9)
Energy intake (kcal/day)	2108	(570)
Protein intake (g/day)	75.4	(22.6)
Fat intake (g/day)	73.0	(22.7)
Carbohydrate intake (g/day)	279.9	(87.6)
Dietary fiber intake (g/day)	12.5	(4.8)
Salt-equivalent intake (g/day)	11.2	(2.7)

BMI, body mass index; SAF, skin autofluorescence; AU, arbitrary units. SD, standard deviation. Values are means (SD).

**Table 2:** SAF levels in participants categorized into two groups according to health-related characteristics.

		Group 1	Group 2	*p*-Value
BMI	Size	*n* = 162	*n* = 153	
	(kg/m^2^)	≤17.9	>17.9	
	SAF	1.08 (1.04–1.11)	1.05 (1.03–1.07)	0.226
Menarche	Size	*n* = 100	*n* = 215	
	(yes/no)	no	yes	
	SAF	1.10 (1.00–1.20)	1.10 (1.00–1.10)	0.134
Food allergy	Size	*n* = 265	*n* = 50	
	(yes/no)	no	yes	
	SAF	1.10 (1.00–1.10)	1.10 (1.00–1.10)	0.128
Hay fever	Size	*n* = 183	*n* = 132	
	(yes/no)	no	yes	
	SAF	1.10 (1.00–1.10)	1.10 (1.00–1.10)	0.573
Weight-loss dieting	Size	*n* = 285	*n* = 30	
	(yes/no)	no	yes	
	SAF	1.10 (1.00–1.10)	1.10 (1.00–1.20)	0.102

BMI, body mass index. BMI groups were defined using the median. SAF, skin autofluorescence. Values are medians (interquartile ranges). *p*-values were calculated using the Mann–Whitney U test.

**Table 3:** SAF levels in participants categorized into four groups according to quartiles of energy or major nutrient intakes.

		Q1	Q2	Q3	Q4	*p* for Trend
Energy	(kcal)	≤1731	>1731, ≤2060	>2060, ≤2411	>2411	
	Size	*n* = 78	*n* = 80	*n* = 79	*n* = 78	
	SAF	1.09 (1.06–1.12)	1.04 (1.01–1.07)	1.07 (1.04–1.10)	1.06 (1.03–1.09)	0.285
Available carbohydrate	(g)	≤210.3	>210.3, ≤257.0	>257.0, ≤302.7	>302.7	
	Size	*n* = 79	*n* = 79	*n* = 79	*n* = 78	
	SAF	1.07 (1.04–1.10)	1.06 (1.03–1.09)	1.06 (1.04–1.09)	1.06 (1.03–1.09)	0.520
Protein	(g)	≤59.9	>59.9, ≤72.7	>72.7, ≤88.4	>88.4	
	Size	*n* = 78	*n* = 80	*n* = 79	*n* = 78	
	SAF	1.06 (1.03–1.09)	1.05 (1.02–1.08)	1.06 (1.03–1.09)	1.08 (1.05–1.11)	0.421
Fat	(g)	≤57.6	>57.6, ≤71.5	>71.5, ≤87.0	>87.0	
	Size	*n* = 78	*n* = 80	*n* = 79	*n* = 78	
	SAF	1.07 (1.04–1.10)	1.06 (1.03–1.09)	1.05 (1.02–1.08)	1.06 (1.03–1.09)	0.390
Vitamin B_1_	(mg)	≤0.67	>0.67, ≤0.81	>0.81, ≤1.01	>1.01	
	Size	*n* = 78	*n* = 80	*n* = 79	*n* = 78	
	SAF	1.06 (1.03–1.09)	1.07 (1.04–1.10)	1.05 (1.02–1.08)	1.07 (1.04–1.10)	0.611
Vitamin B_2_	(mg)	≤1.21	>1.21, ≤1.47	>1.47, ≤1.82	>1.82	
	Size	*n* = 78	*n* = 81	*n* = 79	*n* = 77	
	SAF	1.07 (1.04–1.10)	1.05 (1.03–1.08)	1.05 (1.02–1.08)	1.08 (1.05–1.11)	0.858
Vitamin C	(mg)	≤85.6	>85.6, ≤113.0	>113.0, ≤148.6	>148.6	
	Size	*n* = 78	*n* = 80	*n* = 79	*n* = 78	
	SAF	1.06 (1.03–1.09)	1.06 (1.03–1.09)	1.05 (1.02–1.08)	1.08 (1.05–1.11)	0.396
Retinol activity equivalents	(µg RAE)	≤476.9	>476.9, ≤632.4	>632.4, ≤864.6	>864.6	
	Size	*n* = 79	*n* = 78	*n* = 80	*n* = 78	
	SAF	1.05 (1.02–1.08)	1.07 (1.04–1.10)	1.05 (1.02–1.08)	1.07 (1.05–1.10)	0.510
Calcium	(mg)	≤492.7	>492.7, ≤655.4	>655.4, ≤889.2	>889.2	
	Size	*n* = 79	*n* = 79	*n* = 79	*n* = 78	
	SAF	1.06 (1.03–1.09)	1.06 (1.03–1.09)	1.06 (1.03–1.09)	1.07 (1.04–1.10)	0.652
Salt-equivalent	(g)	≤9.5	>9.5, ≤11.1	>11.1, ≤12.5	>12.5	
	Size	*n* = 78	*n* = 80	*n* = 79	*n* = 78	
	SAF	1.08 (1.05–1.11)	1.04 (1.01–1.07)	1.06 (1.03–1.09)	1.08 (1.05–1.11)	0.907
Dietary fiber	(g)	≤9.35	>9.35, ≤11.7	>11.7, ≤14.5	>14.5	
	Size	*n* = 78	*n* = 80	*n* = 79	*n* = 78	
	SAF	1.05 (1.02–1.08)	1.06 (1.03–1.09)	1.06 (1.03–1.09)	1.08 (1.05–1.11)	0.283

SAF, skin autofluorescence. SAF values are presented as estimated marginal means with 95% Wald CI. *p* for trend was calculated using generalized linear models. After adjusting for potential confounders, no significant trends were observed in SAF across energy or nutrient-intake quartiles.

**Table 4:** SAF levels in participants categorized into four groups according to quartiles of food- group intakes.

		Q1	Q2	Q3	Q4	*p* for Trend
Cereals	(g)	≤336.4	>336.4, ≤416.1	>416.1, ≤522.3	>522.3	
	Size	*n* = 79	*n* = 79	*n* = 79	*n* = 78	
	SAF	1.07 (1.05–1.10)	1.04 (1.01–1.07)	1.07 (1.04–1.10)	1.06 (1.03–1.09)	0.909
Legumes	(g)	≤20.1	>20.1, ≤39.9	>39.9, ≤81.7	>81.7	
	Size	*n* = 73	*n* = 87	*n* = 77	*n* = 78	
	SAF	1.03 (1.00–1.06)	1.07 (1.04–1.10)	1.08 (1.05–1.11)	1.06 (1.03–1.09)	0.118
Meats	(g)	≤60.3	>60.3, ≤74.4	>74.4, ≤98.8	>98.8	
	Size	*n* = 72	*n* = 86	*n* = 81	*n* = 76	
	SAF	1.08 (1.05–1.11)	1.04 (1.02–1.07)	1.04 (1.02–1.07)	1.09 (1.06–1.12)	0.688
Fish and shellfish	(g)	≤33.5	>33.5, ≤49.4	>49.4, ≤74.4	>74.4	
	Size	*n* = 79	*n* = 79	*n* = 79	*n* = 78	
	SAF	1.06 (1.03–1.09)	1.07 (1.04–1.10)	1.04 (1.01–1.07)	1.08 (1.05–1.11)	0.729
Dairy	(g)	≤99.5	>99.5, ≤194.2	>194.2, ≤331.6	>331.6	
	Size	*n* = 79	*n* = 79	*n* = 79	*n* = 78	
	SAF	1.07 (1.04–1.09)	1.08 (1.05–1.10)	1.06 (1.03–1.09)	1.05 (1.02–1.08)	0.382
Green and yellow vegetables	(g)	≤65.9	>65.9, ≤98.6	>98.6, ≤142.6	>142.6	
	Size	*n* = 81	*n* = 77	*n* = 82	*n* = 75	
	SAF	1.05 (1.02–1.08)	1.09 (1.06–1.12)	1.04 (1.01–1.07)	1.07 (1.04–1.10)	0.911
Other vegetables	(g)	≤83.9	>83.9, ≤130.2	>130.2, ≤182.4	>182.4	
	Size	*n* = 79	*n* = 78	*n* = 79	*n* = 79	
	SAF	1.04 (1.01–1.07)	1.06 (1.03–1.09)	1.07 (1.04–1.10)	1.07 (1.05–1.10)	0.107
Fruits	(g)	≤38.5	>38.5, ≤88.5	>88.5, ≤154.5	>154.5	
	Size	*n* = 79	*n* = 79	*n* = 79	*n* = 78	
	SAF	1.06 (1.02–1.08)	1.06 (1.03–1.09)	1.07 (1.03–1.09)	1.06 (1.05–1.11)	0.965
Confectioneries	(g)	≤31.5	>31.5, ≤56.2	>56.2, ≤87.6	>87.6	
	Size	*n* = 79	*n* = 79	*n* = 79	*n* = 78	
	SAF	1.06 (1.03–1.09)	1.06 (1.04–1.09)	1.07 (1.04–1.10)	1.06 (1.03–1.09)	0.824
Beverages including tea, coffee, and soft drinks	(g)	≤200.4	>200.4, ≤561.7	>561.7, ≤692.7	>692.7	
	Size	*n* = 78	*n* = 80	*n* = 75	*n* = 82	
	SAF	1.05 (1.02–1.08)	1.07 (1.04–1.09)	1.08 (1.04–1.11)	1.06 (1.03–1.09)	0.639
Egg *	(g)	≤46.5	>46.5			
	Size	*n* = 166	*n* = 149			
	SAF	1.10 (1.00–1.10)	1.00 (1.00–1.10)			0.145

SAF, skin autofluorescence. SAF values are presented as estimated marginal means with 95% Wald CI. *p* for trend was calculated using generalized linear models. After adjusting for potential confounders, no significant trends were observed in SAF across food-group intake quartiles. * For egg intake, quartile categorization was not feasible due to many identical values (BDHQ-15y algorithm); therefore, participants were dichotomized at the median and SAF values were compared using the Mann–Whitney U test (*p*-value shown).

**Table 5:** SAF levels in participants categorized into four groups according to their responses to each lifestyle questionnaire item.

		Group 1	Group 2	Group 3	Group 4	*p* for Trend
Breakfast frequency	(days/w)	7	5–6	3–4	≤2	
	Size	*n* = 288	*n* = 15	*n* = 6	*n* = 6	
	SAF	1.06 (1.04–1.07)	1.08 (1.01–1.15)	1.10 (1.00–1.22)	1.15 (1.04–1.27)	0.121
Fullness at meals	(degree)	Always full	Sometimes full	About 80% full	Less than 80% full	
	Size	*n* = 52	*n* = 148	*n* = 99	*n* = 16	
	SAF	1.08 (1.04–1.11)	1.05 (1.03–1.07)	1.07 (1.04–1.10)	1.07 (1.01–1.14)	0.958
Timing of snack intake *	(timing)	Immediately after returning home	Before dinner	After dinner	Before bedtime	
	Size	*n* = 180	*n* = 94	*n* = 28	*n* = 3	
	SAF	1.06 (1.04–1.08)	1.06 (1.04–1.09)	1.09 (1.04–1.14)	1.10 (0.95–1.27)	0.546
Bowel movement frequency	(days/w)	1–2	3–4	5–6	7	
	Size	*n* = 39	*n* = 107	*n* = 103	*n* = 65	
	SAF	1.05 (1.01–1.09)	1.08 (1.05–1.10)	1.05 (1.03–1.08)	1.06 (1.03–1.09)	0.951
Weekday sleep duration **	(hour)	≤7.0	>7.0, ≤7.75	>7.75, ≤8.33	>8.33	
	Size	*n* = 110	*n* = 77	*n* = 75	*n* = 53	
	SAF	1.07 (1.04–1.09)	1.09 (1.06–1.12)	1.05 (1.02–1.08)	1.03 (0.99–1.06)	0.019
	SAF_1_	1.07 (1.04–1.09)	1.09 (1.06–1.12)	1.05 (1.02–1.08)	1.03 (0.99–1.06)	0.017
	SAF_2_	1.10 (1.07–1.14)	1.12 (1.08–1.16)	1.08 (1.04–1.12)	1.06 (1.02–1.10)	0.018
Subjective sleep quality (perceived restorative sleep)	(degree)	Very good	Good	Not very good	Not rested at all	
	Size	*n* = 34	*n* = 146	*n* = 118	*n* = 17	
	SAF	1.03 (0.99–1.08)	1.05 (1.03–1.07)	1.07 (1.05–1.10)	1.11 (1.05–1.18)	0.033
	SAF_1_	1.03 (0.99–1.08)	1.05 (1.03–1.08)	1.07 (1.05–1.10)	1.11 (1.05–1.18)	0.041
	SAF_2_	1.06 (1.01–1.11)	1.09 (1.05–1.12)	1.11 (1.07–1.14)	1.13 (1.07–1.20)	0.048
Exercise frequency	(days/w)	6–7	3–5	1–2	<1	
	Size	*n* = 43	*n* = 104	*n* = 79	*n* = 89	
	SAF	1.09 (1.05–1.13)	1.07 (1.05–1.10)	1.07 (1.04–1.10)	1.03 (1.00–1.06)	0.021
	SAF_1_	1.09 (1.05–1.13)	1.07 (1.05–1.10)	1.07 (1.04–1.10)	1.03 (1.00–1.06)	0.019
	SAF_2_	1.12 (1.07–1.17)	1.10 (1.07–1.14)	1.10 (1.06–1.14)	1.06 (1.01–1.10)	0.019

SAF, skin autofluorescence. SAF values are presented as estimated marginal means (95% Wald CI). *p* for trend was calculated using generalized linear models. * For snack timing, 10 participants without snacking habits were excluded (*n* = 305). ** Participants were categorized into four groups based on responses to each question (4-point scale); sleep duration was categorized into quartiles. SAF, crude (Model 0). SAF_1_, adjusted for energy intake and available carbohydrate intake (Model 1). SAF_2_, additionally adjusted for BMI, menarche status, food allergy, hay fever, and weight-loss dieting status (Model 2). For brevity, adjusted results are presented only for factors with significant linear trends.

## Data Availability

The data presented in this study are available on request from the corresponding author. The data are not publicly available due to ethical restrictions and privacy concerns involving minors.

## Abbreviations

The following abbreviations are used in this manuscript:

AGE	Advanced Glycation End Product
AU	Arbitrary Units
BMI	Body Mass Index
CI	Confidence Interval
GLM	Generalized Linear Model
HOMA-IR	Homeostatic Model Assessment of Insulin Resistance
MHLW	Ministry of Health, Labour and Welfare
PSQI	Pittsburgh Sleep Quality Index
SAF	Skin Autofluorescence
SD	Standard Deviation
